# RAGE Deficiency Impairs Bacterial Clearance in Murine Staphylococcal Sepsis, but Has No Significant Impact on Staphylococcal Septic Arthritis

**DOI:** 10.1371/journal.pone.0167287

**Published:** 2016-12-01

**Authors:** Majd Mohammad, Manli Na, Amanda Welin, Mattias N. D. Svensson, Abukar Ali, Tao Jin, Rille Pullerits

**Affiliations:** Department of Rheumatology and Inflammation Research, Institute of Medicine, The Sahlgrenska Academy at University of Gothenburg, Gothenburg, Sweden; University of Pittsburgh, UNITED STATES

## Abstract

**Background:**

Septic arthritis is a serious joint disease often caused by *Staphylococcus aureus (S*. *aureus)*. Receptor for Advanced Glycation End products (RAGE) has an important role in several infections. We sought to investigate the role of RAGE in staphylococcal septic arthritis and sepsis in mice.

**Methods:**

Wild-type (WT) and RAGE deficient (RAGE^-/-^) mice were intra-articularly or intravenously inoculated with an arthritic or septic dose of *S*. *aureus* LS-1 strain. Clinical arthritis, weight development and mortality were monitored for 14 days. Serum levels of cytokines, kidney bacterial loads as well as micro-CT and histopathology of the joints were assessed.

**Results:**

RAGE^-/-^ mice with septic arthritis had significantly lower IL-17A and higher bone mineral density (BMD) compared to the control group. However, no significant differences between the groups were observed regarding the weight loss, the severity and frequency of arthritis, and bacterial loads in the kidneys. In mice with sepsis, the overall mortality rate was similar in RAGE^-/-^ (39%) and in WT mice (45%). However, RAGE^-/-^ mice with sepsis had significantly higher bacterial load in their kidneys compared to the WT controls. In line with data from hematogenous *S*. *aure*us arthritis, RAGE deficiency had no impact on arthritis severity in local joint infection.

**Conclusions:**

Our results indicate that lack of RAGE has no significant impact on septic arthritis. However, RAGE^-/-^ mice had significantly higher BMD compared to WT mice, which coincided with lower IL-17A in RAGE^-/-^ mice. In sepsis, RAGE deficiency impairs bacterial kidney clearance.

## Introduction

*Staphylococcus aureus* (*S*. *aureus*) is a gram-positive bacterium that usually colonizes humans, mostly in the anterior nares and the skin. Around 80% of the human population is carriers of *S*. *aureus*, of which 30% are persistently colonized [1]. Due to its unique structure and its ability to evade the human immune system, *S*. *aureus* is a highly pathogenic bacterium, with the ability to cause a broad range of diseases in humans. Septic arthritis is considered as one of the most aggressive joint diseases and *S*. *aureus* is the most prevalent cause of this severe disease. The joint damage caused by septic arthritis is often irreversible, even when immediate treatment is initiated [2]. More importantly, for those who survive, more than half will have permanent joint dysfunction severely affecting their daily life [3]. The prevalence of septic arthritis in the general population is estimated to 4–10 cases per 100 000 individuals per year [4]. However, the frequency of septic arthritis is 10 times higher in patients with pre-existing joint diseases [4]. Sepsis, a more severe form of infection often also caused by staphylococci, corresponds to a high mortality rate in the western world, approaching 15–20% [5] and is considered as one of the main causes of mortality worldwide [6].

Receptor for advanced Glycation End products (RAGE) is a receptor known to have an important role in inflammation and infection. RAGE is a trans-membrane receptor that belongs to the immunoglobulin superfamily of cell surface molecules [7]. The receptor consists of three immunoglobulin-like regions crucial for ligand binding, a single trans-membrane domain and a cytosolic tail. The cytosolic tail is vital for intracellular signaling of the RAGE receptor [7]. RAGE is localized and expressed on a wide variety of cells, such as monocytes/macrophages, T-cells, endothelial cells, vascular smooth muscle cells, glomerular epithelial cells and neurons [8, 9]. Up-regulation of RAGE occurs in inflammation, diabetes and neurodegenerative disorders, such as Alzheimer's disease, and is a result of enhanced levels of its ligands [9–11]. Although RAGE was first described as a receptor for "advanced glycation end products" (AGEs) [12], it is now known as a multi-ligand receptor interacting with several different ligands [13]. Apart from AGEs, other pro-inflammatory ligands of RAGE include an endogenous danger signal high mobility group box protein 1 (HMGB1) and S100/calgranulin proteins as well as beta2-integrin Mac-1 [14, 15].

The soluble form of the receptor (sRAGE) has been hypothesized to bind to RAGE ligands thus resulting in membrane bound RAGE remaining unoccupied. Therefore, the pro-inflammatory activities associated with these ligands probably will be blocked [16].

RAGE has been targeted in different bacterial infections such as sepsis and pneumonia. Recently, Achouiti *et al* showed that while inducing pneumonia in a mouse model with *S*. *aureus*, RAGE and one of its ligands, HMGB1, have the ability to contribute to lung injury [17]. In addition, van Zoelen *et al* revealed that RAGE facilitated and enhanced the bacterial levels in the lungs, which resulted in an impaired host response in a pneumococcal pneumonia mouse model [18]. The role of RAGE has also been investigated in animal sepsis models [19]. In the sepsis model caused by cecal ligation and puncture, the mortality rate was significantly lower in RAGE deficient mice compared to wild-type mice indicating the protective role of RAGE deficiency in the host response [19]. Moreover, a recent animal study with LPS-induced septic shock indicated that not only RAGE deficient mice but also wild-type mice treated with sRAGE had significantly longer survival compared to the untreated groups [20].

Despite intense treatment with bactericidal antibiotics, approximately 50% of the septic arthritis patients do not regain their joint function [3]. The response from the immune system seems to contribute to the joint destruction due to its overwhelming activity even when the microorganisms have been eradicated [4, 21]. Intriguingly, Ali *et al* recently showed that antibiotic killed *S*. *aureus*, while injected intra-articularly into the knee joints of healthy mice, could induce and maintain arthritis [22]. In this study, RAGE deficient mice displayed significantly decreased synovitis incidence and severity compared to wild-type counterparts indicating the partial involvement of RAGE in dead *S*. *aureus* induced joint inflammation [22].

The aim of this study was to identify the role of RAGE in the immune response in staphylococcal septic arthritis. More specifically, we sought to determine whether RAGE signaling affects the clinical and histopathological outcome of septic arthritis. In addition, the impact of RAGE deficiency on staphylococcal infection using higher doses of bacteria (staphylococcal sepsis) was also studied.

## Materials and Methods

### Mice

C57Bl/6 WT mice of both sexes were purchased from Charles River Laboratories (Sulzfeld, Germany). RAGE^-/-^ mice, backcrossed over 10 times to a C57Bl/6 background, were kindly provided by Prof. Arnold, Deutsches Krebsforschungszentum and Prof. Nawroth, University Clinical Centre of Heidelberg, Germany [23, 24]. The mice were bred and housed in the animal facility of the Department of Rheumatology and Inflammation Research, University of Gothenburg. All mice were fed with standard chow and water *ad libitium* under standard conditions of temperature and light and used for the experiments at the age of 6–10 weeks. The ethical committee of animal research of Gothenburg approved the study.

### Experimental protocol for staphylococcal septic arthritis and sepsis

For induction of staphylococcal septic arthritis and sepsis in mice, *S*. *aureus* strain LS-1 was used and prepared as previously described [25]. Before experiments, the bacterial solution was thawed, washed in PBS, and adjusted to the concentration required.

To study the effect of RAGE on staphylococcal septic arthritis, three separate *in vivo* experiments were performed using WT mice (n = 9-10/experiment) and RAGE^-/-^ mice (n = 9–10 mice/experiment). The mice received a dose of 1.0–1.2 x 10^7^ colony forming units (CFUs) LS-1 *S*. *aureus* intravenously (i.v.) in the tail vein and were monitored individually. All experiments were done in the same manner and terminated on day 14. The data from three experiments were pooled.

To study the impact of RAGE on staphylococcal sepsis, two independent experiments were carried out with altogether 20 WT and 28 RAGE^-/-^ mice. Each mouse was inoculated i.v. with a dose of 6.2–6.6 x 10^7^CFU *S*. *aureus* LS-1 strain and the survival of all mice were monitored every 8 hours. Mice were sacrificed if deemed not to survive until the next scheduled time point and considered dead due to sepsis. The two sepsis experiments were performed equally and were terminated on day 14.

To study the effect of RAGE on local staphylococcal joint infection, different doses of *S*. *aureus* LS-1 strain were injected intra-articularly into the knee joints of WT and RAGE^-/-^ mice. A low dose of bacteria (1.7x10^2^ CFU) on WT mice (n = 10) and RAGE^-/-^ mice (n = 17) and a high dose of bacteria (14x10^2^ CFU) in WT mice (n = 4) and RAGE^-/-^ mice (n = 9) were injected into the knee joints. The mice were sacrificed at day 4 and the joints evaluated histologically.

### Clinical evaluation of septic arthritis mice

Clinical arthritis and weight was evaluated on days 3, 5, 7, 10, 12 and 14 post infections. To minimize bias, an observer (T.J) was tasked with the evaluation in a blinded manner and a scoring system of 0–3 points was used for each limb. The scoring system as described previously [26, 27] was following: 1—mild swelling and/or erythema, 2—moderate swelling and/or erythema and 3—major swelling and/or erythema. Each mouse was evaluated individually and could be awarded 12 points as maximum.

### Collection of blood and tissue samples

Anesthesia with ketamine hydrochloride (Pfizer AB, Sweden) and metedomidine (Orion Pharma, Finland) was performed on all the mice before the blood was collected and the mice were then sacrificed. The blood samples were centrifuged after clot formation 10 minutes at 1500 g, the serum was collected and stored at -20°C until used. The kidneys were aseptically removed, placed into sterile bags and kept on ice. The limbs were removed and placed into 4% formaldehyde solution. The kidneys were homogenized and then up to 5 serial dilutions 1:10 in PBS were performed followed by spreading of 100μl of bacterial suspension onto horse blood agar plates. The plates were cultured for 24 hours at 37°C and the bacteria quantified as CFUs.

### *In vitro* spleen cell stimulation

The spleens from five healthy RAGE^-/-^ and six WT mice were removed under sterile conditions. Each spleen was passed through a nylon mesh and washed with PBS. Ammonium chloride was used to lyse the erythrocytes. The cell suspensions were adjusted to 2x10^6^/mL in Iscove's complete medium (1% L-glutamin, 5x10^-5^ M mercaptoethanol, 10% heat inactivated fetal calf serum (Integro B.V.; Leuvenheim, The Netherlands) and 50 μg/mL gentamycin), added to a 96-well plate, incubated at 37°C for 2 hours and stimulated 72 hours with toxic shock syndrome toxin 1 (TSST-1) 100 ng/mL, LPS 1 μg/mL, heat-killed bacteria 1x10^8^ bacteria/mL or Iscove's medium (negative control). 1μCi [^3^H]thymidine (Amersham, Bucks, UK) was added for incorporation 12 hours before the cells were harvested and the proliferative response read with a micro-β counter.

### Cytokines measurements

Cytometric Bead array (CBA) Mouse Th1/Th2/Th17 Cytokine Kit (BD Biosciences) was used according to manufacturer’s instructions to determine the serum levels of tumor necrosis factor alpha (TNF-α), interferon-gamma (IFN-γ), interleukin-6 (IL-6), interleukin-4 (IL-4), interleukin-2 (IL-2), interleukin-17 (IL-17) and interleukin-10 (IL-10) from mice. Analysis was performed with a FacsCanto2 flow cytometer and the data analyzed using FCAP array software (BD Biosciences). The cytokines were measured in the serum from infected WT (n = 27) and RAGE^-/-^ (n = 28) mice as well as from uninfected female WT (n = 6) and RAGE^-/-^ (n = 10) mice. The limits of detection for cytokines were as follows: IL-2 0.1 pg/ml, IL-4 0.03 pg/ml, IL-6 1.4 pg/ml, IFN-γ 0.5 pg/ml, TNF-α 0.9 pg/ml, IL-17A 0.8 pg/ml and IL-10 16.8 pg/ml.

### Micro-computed tomography (micro-CT)

Imaging of the joints of mice was performed *ex vivo* with micro-CT scanning to detect bone destruction after the studies were terminated. Knee-, hip-, ankle-, shoulder-, elbow-, and wrist joints were examined with a Skyscan1176 micro-CT (Bruker, Antwerp, Belgium) with the settings adjusted to a voxel size of 18 μm, an aluminium filter of 0.2-mm, exposure time of 29 ms conducted at 45 kV/555 μA. The X-ray projections were set at an interval of 0.5° with a scanning rotation of 180°. NRECON software (version 1.5.1; Bruker) was afterwards used to reconstruct the projection images into 3D images followed by analyzing with CT-vox (version 2.4; Bruker). An experienced observer (T.J) evaluated in a blinded manner the extent of bone and cartilage destruction on a scale grade from 0–3 [28]. For calculation of the bone mineral density (BMD), calibration of the Skycan CT system was performed as previously described [29].

### Histopathology analysis

The limbs were stored in 4% formaldehyde, decalcified for 30 hours in Histolab Parengy's^®^ decalcification solution, followed by dehydration and then embedded in paraffin. Afterwards 5 μm thick samples were cut with a microtome, transferred to a glass slide and stained with hematoxylin and eosin. The slides were coded and evaluated in a blinded manner by two observers to assess erosion and synovial hypertrophy with a scoring range of 0–3 points, as previously described [22, 27].

### Statistical analysis

For statistical analysis, GraphPad Prism version 6 for Windows (GraphPad software) was used. To compare the differences between the groups, Mann-Whitney U test and Fischer's exact test were used and all results are reported as mean ± standard error of the mean (SEM) unless indicated otherwise. The P <0.05 was considered statistically significant.

## Results

### RAGE deficient mice present similar clinical severity and frequency of septic arthritis but impaired bacterial clearance as compared to WT counterparts

**Fig 1:**
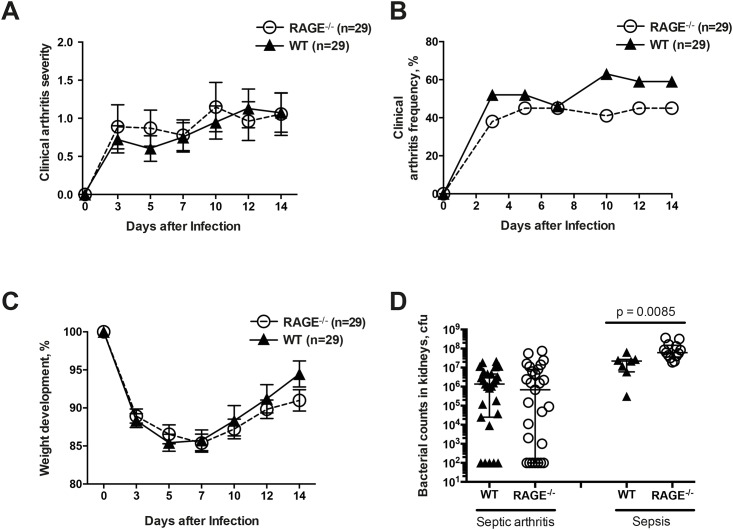
RAGE deficiency did not influence the clinical severity and frequency of septic arthritis but impaired bacterial clearance in staphylococcal sepsis. The clinical severity of arthritis (A), the frequency of arthritis (B), and changes in the body weight (C) in Receptor for Advanced Glycation End products deficient (RAGE^-/-^) and wild-type (WT) mice after intravenous infection with *S*. *aureus* LS-1 strain. The data are reported as mean ± SEM and analyzed with Mann-Whitney U test and Fisher's exact test. Persistence of *S*. *aureus* in the kidneys (D) of WT mice (n = 27) and RAGE^-/-^ mice (n = 27) at the end of septic arthritis experiments as well as in WT mice (n = 7) and RAGE^-/-^ mice (n = 13) surviving the sepsis experiment. The results of the bacterial count are expressed as colony forming units (CFU) and the data are reported as median and interquartile range (IQR).

To investigate whether the lack of RAGE affects the frequency and severity of clinical arthritis, RAGE^-/-^ and WT mice were intravenously inoculated with an arthritic dose of *S*. *aureus*. The clinical arthritis severity and the arthritis frequency were evaluated every 2–3 days. Mice from both groups developed arthritis rapidly within first days after infection. The mean arthritis severity reached its peak on day 10 for RAGE^-/-^ mice and on day 12 for its WT counterpart being almost identical in both groups at the end of experiment (1.06 ± 0.28 in RAGE^-/-^ and 1.07 ± 0.26 in WT mice, respectively)(Fig 1A). The overall frequency of septic arthritis on day 14 was evident in 45% of RAGE^-/-^ mice and in 59% of controls. Although there was a trend towards higher clinical arthritis frequency in the WT mice, the differences between groups were not significant (Fig 1B).

In our septic arthritis animal model, weight decrease is a parameter that reflects the health status of the mice. The change in weight loss did not show any significant difference between the groups (Fig 1C). The mice started to lose weight immediately after infection and by day 5, RAGE^-/-^ had lost 13% of its total body weight compared to 15% of the WT mice. Both groups started to recover their weight from day 7 and at the end of the experiment RAGE^-/-^ mice had recovered up to 91% and WT mice up to 95% of its original body weight.

In addition, the bacterial clearance in the kidneys of infected mice was assessed. The median bacterial load in the kidneys that reflects the ability of the host to clear the bacteria was lower in RAGE^-/-^ mice compared to WT mice (median 6.9x10^5^; interquartile range (IQR) 1.0.x102–8.0x10^6^ CFU versus 14x10^5^; IQR 2.5x10^4^–4.6x10^6^ CFU, respectively) in the septic arthritis experiment (Fig 1D). However, no statistical differences between the groups were observed. In terms of the sepsis experiment, the median bacterial load resulted in significantly higher bacterial counts in the RAGE^-/-^ group compared to the WT group (median 6.1x10^7^; IQR 3.6x10^7^ – 15x10^7^ CFU versus 2.2x10^7^; IQR 0.6x10^7^–2.7x10^7^ CFU, respectively, p = 0.0085)(Fig 1D).

### RAGE deficiency has no impact on the radiological and histopathological joint destruction in septic arthritis

**Fig 2:**
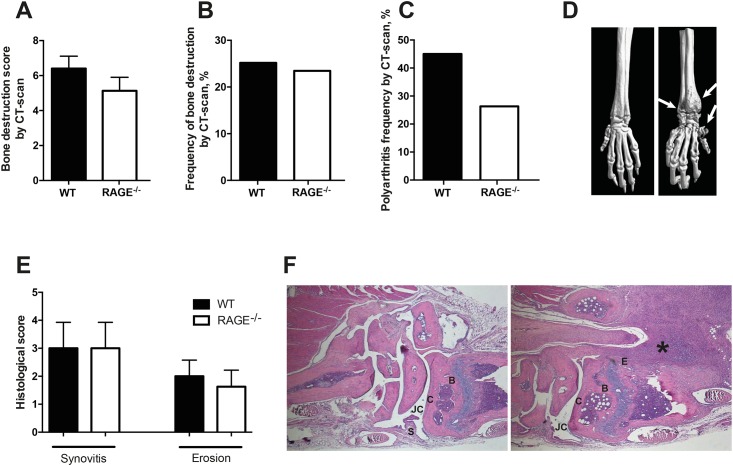
RAGE deficient mice with septic arthritis presented similar radiological and histopathological joint destructions as wild-type mice. The radiological accumulative bone destruction scores (A), the frequency of bone destructions (B) and the frequency of polyarthritis (C) in WT and RAGE^-/-^ mice as evaluated by micro-CT scan. The data are reported as mean ± SEM and analyzed with Mann-Whitney U test and Fisher's exact test. Representative micro-CT scan images (D) of wrist joints from RAGE^-/-^ mice illustrating a healthy joint (left panel) and a joint with severe septic arthritis (right panel). The erosions are indicated by the arrows. Histopathologic evaluation of synovitis and bone erosions (E) in the joints from all 4 limbs of WT mice (n=7) and RAGE^-/-^ mice (n=8) on day 14 post-infection. The data are reported as mean ± SEM and analyzed with Mann-Whitney U test. Illustrative photomicrographs (F) showing a healthy wrist joint from RAGE^-/-^ mouse (left panel) and a heavily inflamed wrist joint with severe bone and cartilage destruction from WT mouse (right panel). Histologic staining was performed using hematoxylin and eosin. The asterisk indicates an inflamed synovium. Abbreviations: B, bone; C, cartilage; E, erosion of bone and cartilage; JC, joint cavity; S, synovial tissue. Original magnification × 25.

In order to obtain further information regarding severity of septic arthritis in joints from the mice, micro-CT was used. In this way, the destructions of the clinically not accessible joints could be verified. All joints including hip-, knee-, ankle-, shoulder-, elbow-, and wrist joints from both sides of each mouse were examined. We did not find any significant difference between the RAGE^-/-^ and WT groups. The mean accumulative bone destruction score, as assessed by evaluating 12 joints per mouse, was 6.4 ± 0.8 for WT mice and 5.1 ± 0.7 for RAGE^-/-^ mice (Fig 2A and 2D). Twenty five percent of joints from WT mice developed radiological bone destructions as compared to 23% in RAGE^-/-^ mice (Fig 2B). The frequency of polyarthritis i.e. arthritis in 4 or more joints was also assessed and corresponded to 26% in RAGE deficient mice compared to 45% in control mice (Fig 2C).

In addition, a more detailed analysis was performed in order to investigate which particular joints were affected in the septic arthritis mice. Although no significant differences between WT and RAGE^-/-^ mice occurred in terms of severity and frequency in the sub-group joints, 40% of all shoulders in WT mice displayed signs of bone destruction compared to 24% in RAGE^-/-^ mice. In contrast, 53% of all knee joints in RAGE^-/-^ mice exhibited signs of bone destruction compared to 30% in WT controls (p = 0.06) (Table 1).

**Table 1:** Subgroup analysis of bone destruction by a micro-CT scan.

	Severity (Mean ± SEM)		Frequency (%)	
	WT	RAGE^-/-^	WT	RAGE^-/-^
Hands	0.7 ± 0.2	0.4 ± 0.1	33	21
Elbows	0.08 ± 0.06	0.05 ± 0.03	3	3
Shoulders	0.8 ± 0.1	0.5 ± 0.1	40	24
Hips	0.3 ± 0.1	0.3 ± 0.1	21	11
Knees	0.6 ± 0.1	1.0 ± 0.2	30	53
Feet	0.6 ± 0.1	0.4 ± 0.1	25	26

The data are reported as mean ± SEM.

Joint sections from the limbs were also analyzed histologically with regard to synovial inflammation as well as bone erosions. The mean synovitis score was identical for both groups (3.0 ± 0.9 for both RAGE^-/-^ and WT mice) and the mean erosion score did not differ from WT mice (2.0 ± 0.6) compared to RAGE^-/-^ mice (1.6 ± 0.6)(Fig 2E and 2F).

### RAGE deficiency does not aggravate the arthritis severity in local staphylococcal knee joint infection

**Fig 3:**
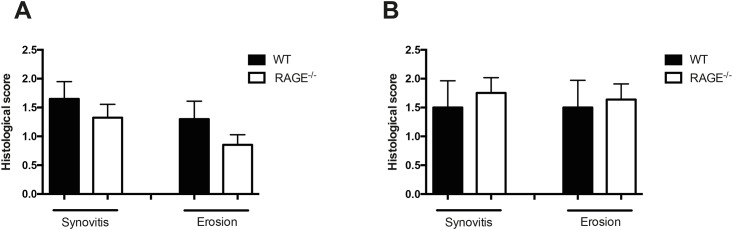
Local staphylococcal joint infection resulted in a similar outcome on RAGE deficient mice as compared to controls. Histopathologic evaluation of synovitis and bone erosions scores following intra-articular injection of *S*. *aureus* into the knee joints of mice. The WT and RAGE^-/-^ mice were injected with a low dose of bacteria (1.7x10^2^ CFU) (A) or a high dose of bacteria (14x10^2^ CFU) (B). The data are reported as mean ± SEM and analyzed with Mann-Whitney U test.

To study the effect of RAGE on local staphylococcal joint infection, WT and RAGE^-/-^ mice were injected either with a low dose (1.7x10^2^ CFU) or a high dose (14x10^2^ CFU) of *S*. *aureus* LS-1 strain intra-articularly into the knee joints. The histological analysis of knee joints injected with lower dose of bacteria at day 4 showed no difference between RAGE^-/-^ mice and its WT counterparts regarding the mean synovitis score (1.7 ± 0.3 in WT and 1.3 ± 0.2 in RAGE^-/-^ mice) or mean erosion score (1.3 ± 0.3 in WT and 0.9 ± 0.2 in RAGE^-/-^ mice), respectively (Fig 3A). Similarly, exposure to a higher dose of *S*. *aureus* in WT controls and RAGE^-/-^ mice did not give rise to any differences between the groups, resulting in a mean synovitis score of 1.5 ± 0.5 in WT compared to 1.8 ± 0.3 in RAGE^-/-^ mice as well as a mean erosion score of 1.5 ± 0.5 in WT compared to 1.6 ± 0.3 in RAGE^-/-^ mice (Fig 3B).

### RAGE^-/-^ mice present less bone loss in septic arthritis as compared to WT counterparts

**Fig 4:**
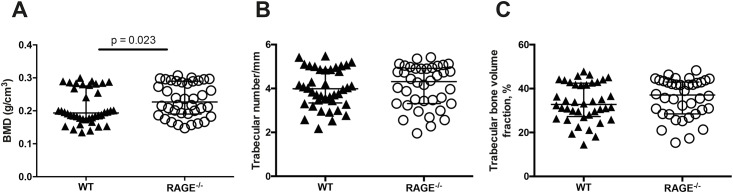
RAGE deficiency led to less bone loss in mice with septic arthritis. A graph showing quantitative evaluation of the bone mineral density (BMD) (A), the trabecular number (B) and the percentage of trabecular bone volume fraction (C) in both of the tibial metaphysis from WT mice (n = 20) and RAGE^-/-^ mice (n = 19) measured using micro-CT. The data are reported as median and IQR and analyzed with Mann-Whitney U test.

To study whether RAGE deficiency has any possible impact on the bone mineral density in septic arthritis, BMD was measured in RAGE^-/-^ and WT mice with septic arthritis using a micro-CT scan. The BMD was measured in the tibia as previously described [29]. Healthy WT and RAGE^-/-^ mice did not have any differences in BMD. Septic arthritis WT mice had significantly lower BMD compared to RAGE^-/-^ mice (p = 0.023)(Fig 4A).

In addition, the trabecular number as well as the percentage of trabecular bone volume fraction was also analyzed. However, no significant differences between the groups were found (Fig 4B and 4C).

### RAGE^-/-^ mice display decreased levels of circulating IL-17A in septic arthritis whereas healthy RAGE^-/-^ mice have increased levels of circulating pro-inflammatory cytokines

**Fig 5:**
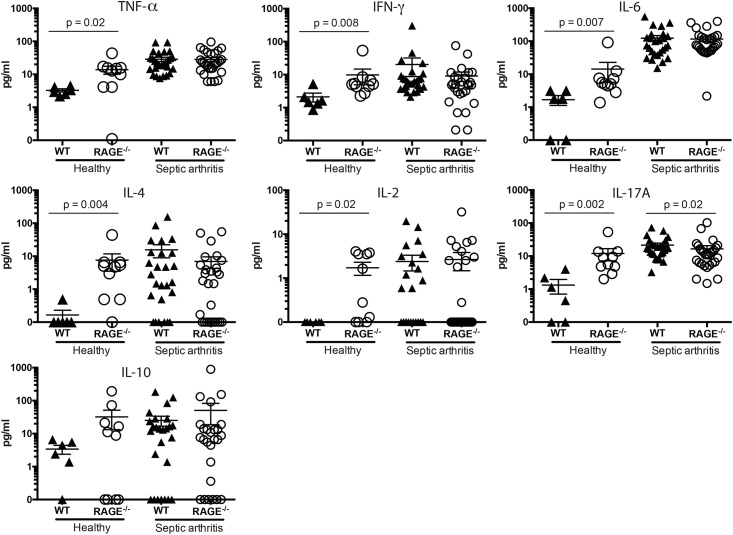
RAGE deficient mice displayed decreased levels of circulating IL-17A in septic arthritis whereas healthy mice had increased levels of several pro-inflammatory cytokines. Serum levels of tumor necrosis factor α (TNF-α), interferon γ (IFN-γ), interleukin 6 (IL-6), interleukin 4 (IL-4), interleukin 2 (IL-2), interleukin 17A, (IL-17A) and interleukin 10 (IL-10) were determined in healthy uninfected WT and RAGE^-/-^ mice and in mice with septic arthritis at the end of experiment on day 14. The data are reported as mean ± SEM and analyzed with Mann-Whitney U test.

Since cytokines plays a vital role in the immune and inflammatory host response, we investigated the cytokine-chemokine response to systemic staphylococcal infection. The serum cytokine levels of TNF-α, IFN-γ, IL-6, IL-4, IL-2 and IL-10 at the end of septic arthritis experiment on day 14 was measured and did not differ between the infected groups. However, the systemic IL-17A levels were significantly lower in infected RAGE^-/-^ mice (mean 16.6 ± 4.0 pg/mL, p < 0.02) compared to infected WT mice (mean 21.4 ± 3.2 pg/mL)(Fig 5). Interestingly, healthy RAGE^-/-^ mice had significantly higher levels of several serum cytokines (TNF-α, IFN-γ, IL-6, IL-4, IL-2 and IL-17A) compared to healthy WT controls (Fig 5).

### RAGE deficient mice present similar proliferative responses of spleen cells to staphylococcal stimuli compared with WT controls

**Fig 6:**
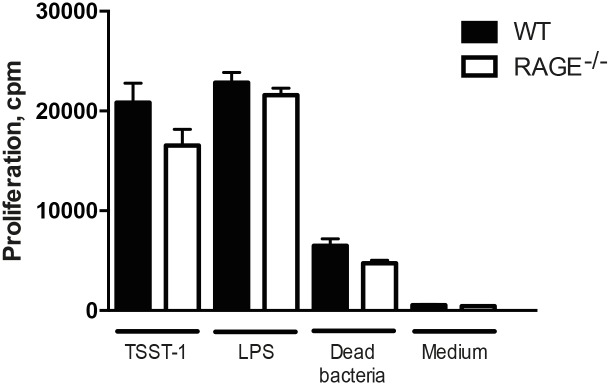
RAGE deficient mice present similar proliferative responses of spleen cells to staphylococcal stimulations as compared to WT controls. The proliferative responses of spleen cells from WT mice (n = 6) and RAGE^-/-^ mice (n = 5) after stimulation with 100 ng/ml of toxic shock syndrome toxin 1 (TSST-1), 1 μg/ml of LPS, 1x10^8^ bacteria/ml of heat-killed *S*. *aureus* and culture medium as negative control. The data are reported as mean ± SEM and analyzed with Mann-Whitney U test.

Next, we sought to study the immune response *in vitro* by using splenocytes from healthy RAGE^-/-^ mice and WT controls. Spleen cell cultures were prepared and stimulated with dead *S*. *aureus* and staphylococcal TSST-1. However, there was no statistical significance between the two groups (Fig 6).

### RAGE deficiency does not affect the survival of mice in staphylococcal septic arthritis and sepsis

**Fig 7:**
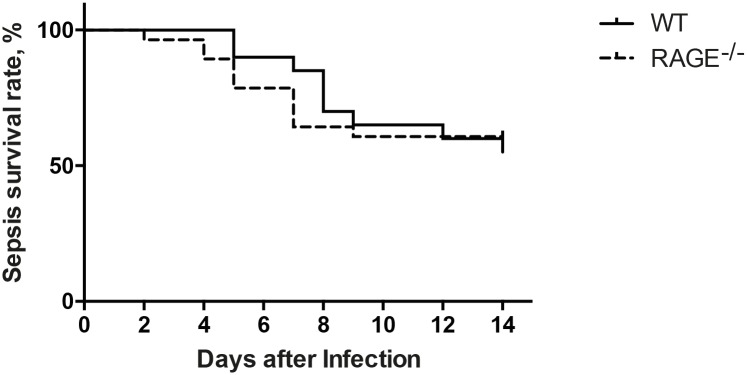
RAGE deficiency did not affect the survival of mice in staphylococcal sepsis. Log rank survival curve showing survival rate comparison between WT mice (n = 20) and RAGE^-/-^ mice (n = 28). The mice were intravenously inoculated with a dose of 6.2–6.6 x 10^7^CFU *S*. *aureus* LS-1 strain and the survival was monitored every 8 hours. The pooled results from two sepsis experiments are shown. Statistical evaluations were performed using the log-rank (Mantel-cox) test.

Intravenous inoculation with arthritic or septic dose of *S*. *aureus* was used to study the role of RAGE in staphylococcal arthritis and sepsis in RAGE^-/-^ and WT mice during the course of 14 days. The overall mortality rate was 3.5% in RAGE^-/-^ mice as compared to 7% in WT mice at the end of septic arthritis experiments (data not shown). In staphylococcal sepsis, the mortality rate was 39% (n = 11) in RAGE^-/-^ group as compared to 45% (n = 9) in WT on day 14 (Fig 7). There was no significant difference in mortality between RAGE^-/-^ and WT mice.

## Discussion

In this study we investigated the effect of RAGE in systemic and local staphylococcal septic arthritis since its role in this infection is unknown. In addition, experiments using higher bacterial doses (staphylococcal sepsis) were carried out as well in order to assess the impact of RAGE with regard to mortality and bacterial load of kidneys.

In our present study, RAGE^-/-^ mice inoculated either with a septic arthritis dose or a sepsis dose of *S*. *aureus* did not differ from WT mice regarding the mortality. As shown by Liliensiek *et al* in a murine model of sepsis caused by cecal ligation and puncture (CLP), the RAGE^-/-^ mice had much higher survival rates compared to WT mice [23]. Furthermore, sRAGE therapy also improved the survival of the mice strongly suggesting that RAGE plays a detrimental role in sepsis and septic shock caused by gram-negative bacteria [23]. The use of anti-murine RAGE monoclonal antibody significantly increased the survival rate of mice compared to mice receiving control serum in another study in CLP murine sepsis model [19]. However, our *S*. *aureus* induced sepsis model differs from the CLP model used by the authors and that could explain our discrepant results regarding the mortality. In the CLP peritonitis model, the peritoneal cavity will be exposed to several mostly gram-negative bacterial strains from fecal material resulting in a severe infection [30]. Therefore, the CLP model causing the sepsis is poly-microbial and not specified to a single type of bacteria, like ours. Also, in our *S*. *aureus* induced septic arthritis and sepsis model, the gram-positive bacteria disseminate via the hematogenous route and directly encounter the bactericidal host defense mechanisms of the blood cells that is different compared to the peritoneal dissemination route. Recently, Achouti *et al* demonstrated that RAGE did not influence the survival of mice in *Streptococcus pneumonia* induced bacteremia that is in corroboration with our results [31]. In their study, the mice were also directly infected with gram-positive bacteria (pneumococci) into the bloodstream.

The results obtained from our experimental studies indicated that there was no difference between the RAGE^-/-^ mice compared to the WT mice regarding both the clinical frequency and the severity of arthritis. The histopathology data as well as the radiological data corresponded well to the clinical arthritis and no significant difference was observed between the groups. In this study, the normal course of the septic arthritis in RAGE^-/-^ and WT mice was followed without intervention. Furthermore, polyarthritis, a more severe form of septic arthritis and an indicator of poor prognosis of the disease, tended to be lower in RAGE^-/-^ mice compared to the WT mice. This, however, did not increase the mortality rate in RAGE^-/-^ mice. In patients, the mortality rate of patients with polyarthritis is 30% despite antibiotics treatment compared to 4% in patients with monoarthritis [32].

RAGE deficient mice displayed significantly more accumulation of bacteria in their kidneys compared to WT mice when given a high septic dose of *S*. *aureus*. When the mice were given a lower, arthritic dose of *S*. *aureus*, we observed no significant differences. It seems that RAGE deficiency deteriorates the ability of mice to successfully eliminate invading bacteria if the bacterial load is high.

All cytokines measured, except of IL-10, in uninfected mice were significantly higher in RAGE^-/-^ mice compared to their WT counterparts. Although no studies regarding this observed phenomenon is available, one could speculate that lack of RAGE somehow affects the expression of cytokines. The underlying mechanism needs further investigation in the future studies. Our results revealed that there was no significant difference regarding TNF-α, IL-2, IFN-γ, IL-4, IL-10 and IL-6 serum cytokine levels between the RAGE^-/-^ mice and the WT mice at the end of septic arthritis experiment. However, IL-17A level was significantly lower in RAGE^-/-^ mice. IL-17A is a potent pro-inflammatory cytokine that is involved in elimination of extracellular bacteria [33]. It induces the production of neutrophil- and macrophage stimulating growth factors and neutrophil-mobilizing chemokines that in turn leads to mobilization of neutrophils into the site of infection. The lower IL-17A levels could possibly contribute to the observed higher bacterial loads in RAGE deficient mice since the recruitment of cells to clear the bacteria might have been affected. However, in a previous study by Henningsson *et al*, differences in terms of severity and frequency of clinical arthritis, mortality and bacterial loads at majority of the time points were not detected between the IL-17A deficient and wild-type mice [33], suggesting that lack of IL-17A during systemic infection with *S*. *aureus* has limited impact.

Previous studies have demonstrated that systemic bone loss rapidly occurred in *S*. *aureus* induced arthritis mice. Both total and trabecular BMD decreased significantly in the infected mice compared to controls [34]. In our study we observed that BMD was significantly lower in *S*. *aureus* infected WT mice compared to RAGE deficient mice. It has been demonstrated in animal models that binding of RAGE with its ligand AGE, contributes to harmful effects in various organs and tissues such as bone and muscle [35]. In fact, this disturbs bone modeling and declines the quality of bone tissue [35, 36] thus causing negative effects on the bone density and mineralization [35, 37]. Indeed, it has been shown that RAGE deficiency has a negative impact on osteoclast function, which resulted in an increased bone mass and BMD as well as reduced bone resorptive activity [38]. Furthermore, a previous study indicated that HMGB1 via RAGE regulates and enhances the osteoclastogenesis, suggesting that RAGE plays a vital role in the HMGB1 mediated osteoclastogenesis *in vivo* [39]. The results from our study also showed that RAGE deficient mice had significantly higher BMD compared to its counterpart, indicating that the presence of RAGE has a negative impact on BMD in mice.

In summary, we conclude that RAGE has a limited impact on onset and on maintenance of staphylococcal septic arthritis in mice, despite decreased IL-17A levels and lower M1- macrophage polarization as compared to wild-type counterparts. However, the bacterial clearance seems to be compromised in RAGE deficient mice, especially with higher bacterial loads. Importantly, BMD was higher in RAGE deficient mice with septic arthritis than in WT mice indicating the role for RAGE in infection induced bone loss.
